# Non-Invasive Evaluation of Patients Undergoing Percutaneous Coronary Intervention for Chronic Total Occlusion

**DOI:** 10.3390/jcm10204712

**Published:** 2021-10-14

**Authors:** Tatsuya Nakachi, Shingo Kato, Naka Saito, Kazuki Fukui, Tae Iwasawa, Tsutomu Endo, Masami Kosuge, Daisuke Utsunomiya, Kazuo Kimura, Kouichi Tamura

**Affiliations:** 1 Department of Cardiology, Saiseikai Yokohamashi Nanbu Hospital, Konandai, Konan-ku, Yokohama 234-0054, Japan; endotu@nanbu.saiseikai.or.jp; 2 Department of Diagnostic Radiology, Yokohama City University Graduate School of Medicine, Fukuura, Kanazawa-ku, Yokohama 236-0004, Japan; shingo.m12226@gmail.com (S.K.); d.utsunomiya@gmail.com (D.U.); 3 Department of Clinical Laboratory, Kanagawa Children’s Medical Center, Mutsukawa, Minami-ku, Yokohama 232-8555, Japan; naka.s.n.5@gmail.com; 4 Department of Cardiology, Kanagawa Cardiovascular and Respiratory Center, Tomioka-higashi, Kanazawa-ku, Yokohama 236-0051, Japan; fukui@kanagawa-junko.jp; 5 Department of Radiology, Kanagawa Cardiovascular and Respiratory Center, Tomioka-higashi, Kanazawa-ku, Yokohama 236-0051, Japan; tae_i_md@wb3.so-net.ne.jp; 6 Division of Cardiology, Yokohama City University Medical Center, Urafune-cho, Minami-ku, Yokohama 232-0024, Japan; masami-kosuge@pop06.odn.ne.jp (M.K.); c-kimura@urahp.yokohama-cu.ac.jp (K.K.); 7 Department of Medical Science and Cardiorenal Medicine, Yokohama City University Graduate School of Medicine, Fukuura, Kanazawa-ku, Yokohama 236-0004, Japan; tamukou@med.yokohama-cu.ac.jp

**Keywords:** chronic total coronary occlusion, percutaneous coronary intervention, speckle-tracking echocardiography, cardiovascular magnetic resonance

## Abstract

Background: As percutaneous coronary intervention (PCI) for chronic total occlusion (CTO) gains wider acceptance as a therapeutic option for coronary artery disease, the importance of appropriate patient selection has increased. Although cardiovascular magnetic resonance imaging (MRI) allows segmental and quantitative analyses of myocardial ischemia and scar transmurality, it has limitations, including contraindications, cost, and accessibility. This study established a non-invasive method to evaluate patients undergoing CTO-PCI using two-dimensional speckle-tracking echocardiography (2D-STE). Methods: Overall, we studied 55 patients who underwent successful CTO-PCI. Cardiovascular MRI and 2D-STE were performed before and 8 ± 2 months after CTO-PCI. Segmental findings of strain parameters were compared with those obtained with late gadolinium enhancement and stress-perfusion MRI. Results: With a cutoff of −10.7, pre-procedural circumferential strain (CS) showed reasonable sensitivity (71%) and specificity (73%) for detecting segments with transmural scar. The discriminatory ability of longitudinal strain (LS) for segments with transmural scar significantly improved during follow-up after successful CTO-PCI in the territory of the recanalized artery (area under the curve (AUC) 0.70 vs. 0.80, *p* < 0.001). LS accuracy was lower than that of CS at baseline (AUC 0.70 vs. 0.79, *p* = 0.048), and was increased at follow-up (AUC 0.80 vs. 0.82, *p* = 0.81). Changes in myocardial perfusion reserve from baseline to follow-up were significantly associated with those in LS but not in CS. Conclusions: Use of 2D-STE may allow the non-invasive evaluation of patients undergoing CTO-PCI to assess the indication before the procedure and treatment effects at follow-up.

## 1. Introduction

Chronic total occlusion (CTO) is commonly encountered among patients referred for coronary angiography, with a reported prevalence of 20–30% [1]. The success rate of percutaneous coronary intervention (PCI) for CTO lesions has improved because of continuous technological evolution [2], integration of techniques based on histopathological assessment [3], and development of treatment algorithms [4,5]. CTO-PCI also has some disadvantages, including unique complications, long procedural times, large contrast volume use, high radiation exposure, and the need for more materials than conventional PCI. Thus, appropriate patient selection is particularly important for CTO-PCI. Scoring systems have been developed to predict the technical results of CTO-PCI [6,7,8], and reports have validated their accuracy [9]. Scoring systems are used for the preprocedural assessment of indications for CTO-PCI, which is only beneficial when used in conjunction with an evaluation of the clinical benefit for the patient.

The clinical benefit of revascularization for CTO depends on the myocardial ischemia status and scar transmurality of the perfusion territory of the CTO lesion. With its excellent spatial resolution, cardiovascular magnetic resonance imaging (MRI) allows us to measure myocardial ischemia quantitatively [10], assess the extent of transmural infarction accurately [11], and predict functional recovery after revascularization in patients with chronic/acute myocardial infarction [11,12]. The cutoff value of the extent of late gadolinium enhancement (LGE) MRI is generally recognized as 50% to detect functional recovery following revascularization [11,13]. However, there are contraindications to cardiovascular MRI (e.g., claustrophobia and implanted metallic devices) and limitations, including cost, accessibility, and gadolinium contrast-related side effects (e.g., nephrogenic systemic fibrosis). Preprocedural, non-invasive methods to assess the CTO-PCI indications are urgently needed.

In contrast, two-dimensional speckle-tracking echocardiography (2D-STE) is a non-invasive method with no contraindications. It provides quantitative measurements of myocardial systolic function more objectively than visual wall motion analysis, with high reproducibility [14].

This study established the use of a non-invasive assessment method using 2D-STE, combined with cardiovascular MRI quantitative measurements of the ischemic burden and the extent of chronic infarction, in patients undergoing CTO-PCI. Therefore, the aim of the present study was two-fold: (1) to determine how echocardiographic strain parameters relate to cardiovascular MRI as a surrogate for viability in decision-making and planning of CTO-PCI, and (2) how to use strain echocardiography as a more objective and sensitive method compared with visual wall motion analysis to identify left ventricular segmental improvements following revascularization.

## 2. Materials and Methods

### 2.1. Patients

This single-center, prospective study included patients with a CTO lesion suitable for recanalization based on coronary angiographic findings. CTO was defined as a coronary obstruction with thrombolysis and myocardial infarction (TIMI) flow grade 0 for >3 months (estimated). All patients had symptomatic angina and/or a positive ischemia study. In patients with multivessel disease, revascularization for non-CTO lesions had to have been achieved. Exclusion criteria included atrial fibrillation, general contraindications to MRI (e.g., device implantation, claustrophobia), contraindication to gadolinium contrast agents (e.g., severe renal dysfunction), contraindication to adenosine administration (e.g., severe asthma, greater than first-degree heart block), insufficient echocardiographic image quality, and significant valvular heart disease. The 16-segment model (American Society of Echocardiography) was used to assess regional systolic function. It divides the left ventricle into six segments for the left anterior descending artery, five for the right coronary artery, and five for the left circumferential artery. Our institutional review board approved this study. All patients provided written informed consent to participate in the study.

### 2.2. Echocardiography

Echocardiographic studies were performed using a Vivid E9 system (GE Vingmed Ultrasound AS, Horten, Norway). Parasternal long-axis and short-axis views (basal, mid-ventricular, apical) and three standard apical views (four-chamber, two-chamber, long axis) were obtained at 56–92 frames/s. Left ventricular (LV) ejection fraction was determined by manually tracing end-systolic and end-diastolic endocardial borders using Simpson’s biplane method. Segmental wall motion was determined on a scale of 1–4: 1, normokinetic; 2, hypokinetic; 3, akinetic; 4, dyskinetic.

### 2.3. Myocardial Strain Assessment

The echocardiographic strain measurements were performed before CTO-PCI and 8 ± 2 months afterward. For circumferential strain (CS) analysis, three short-axis views (basal, mid-ventricular, apical) were analyzed. For longitudinal strain (LS) analysis, three standard apical (2-, 4-, and 3-chamber) views were analyzed according to the 16-segment model. Strain analysis was performed with offline dedicated software (EchoPAC version 113; GE Vingmed Ultrasound AS, Horten, Norway), which allows the analysis of peak systolic segmental CS from short-axis views and of peak global and segmental systolic LS from apical views based on detecting natural acoustic markers. The markers are equally distributed in the myocardium and can be identified and followed frame-to-frame in consecutive images. This system calculates the mean strain values for predefined LV segments. For global strain analysis, peak strain values from each of the 16 segments were averaged. Strain evaluation results were defined by the consensus of two observers. The analytic system automatically determined tracking quality. Segments of suboptimal quality were excluded. End-systole was defined as aortic valve closure in the apical view and was applied to all other views. Ten studies were randomly selected to evaluate intra-observer and inter-observer reproducibility for strain measurements.

### 2.4. Cardiovascular MRI Protocol

Before CTO-PCI and 8 ± 2 months afterward, cardiovascular MRI studies were performed using a 1.5-T MRI scanner (Achieva, Philips Medical Systems, Best, The Netherlands) with 32-channel cardiac coils. After 4 min of adenosine infusion (140 g/kg/min) and 15 min after the intravenous injection of gadolinium diethylenetriamine pentaacetic acid (0.20 mmol/kg) (Magnevist; Schering AG, Berlin, Germany), 8 mm short-axis slices were acquired using a prospective electrocardiogram-gated gradient-echo sequence. Finally, first-pass rest perfusion images were acquired >20 min after stress perfusion imaging.

LGE MRI images and rest–stress first-pass myocardial perfusion MRI images were transferred to a workstation and analyzed with dedicated software (Virtual Place; Aze, Tokyo, Japan). LGE-affected areas were defined as those with signal enhancement ≥ 5 standard deviations from the signal intensity of non-enhanced myocardium. The LGE extent was calculated as the percentage of the contrast-enhanced volume relative to the total myocardial volume of the segment (mass segmental LGE/mass segmental myocardium × 100). Myocardial segments were allocated to three categories [15]: 0%, 1–50%, and 51–100%. For quantitative perfusion analysis, the myocardial perfusion reserve (MPR) was calculated in all CTOs and a remote myocardial territory [10]. MPR was derived from time–intensity curves during first-pass perfusion imaging per segment before and after CTO recanalization. Analysis was according to the Fermi deconvolution function for regional MPR.

### 2.5. Angioplasty Procedure

PCI and stent implantation were performed according to standard practice. Drug-eluting stents were used in all angioplasty procedures. Heparin was administered to maintain an activated clotting time of >250 s. CTO-PCI was performed with modern techniques, such as bilateral injections, specialized hydrophilic, tapered-tip, and stiff wires, parallel-wire techniques using microcatheters or double-lumen catheters, intravascular ultrasonography-guided techniques, and a retrograde approach when possible [2,3]. After the procedure, all patients were prescribed lifelong aspirin plus clopidogrel for at least 12 months. Clinical follow-up was by telephone interview or outpatient visits. Technical success was defined as recanalization and dilation of the target CTO lesion with/without stent implantation, residual stenosis of <30%, and TIMI flow > 2.

### 2.6. Statistical Analysis

Data were statistically analyzed using SPSS Statistics version 24 (IBM, Armonk, NY, USA) and the Medcalc 18.6 statistical program (Medcalc, Ghent, Belgium). Data are expressed as means ± standard deviation. Categorical data are presented as frequencies and percentages. Normality was evaluated using the Shapiro–Wilk test. Changes in echocardiographic parameters between baseline and follow-up were tested using the paired *t*-test for normally distributed values and Mann–Whitney U-test for skewed variables. Receiver operating characteristic (ROC) curves for segmental peak systolic strain were determined to detect segments with transmural LGE > 50% on the preprocedural MRI. ROC analyses and area under the curve (AUC) comparisons were performed according to Delong et al. [16]. Univariate and multivariate linear regression analyses were used to evaluate the relationships between changes in strain parameters, %LGE, and changes in MPR from baseline to follow-up. Values of *p* < 0.05 were considered to indicate statistical significance. Reproducibility of strain measurements was expressed by the intra-class correlation coefficient.

## 3. Results

### 3.1. Patient Characteristics

**Figure 1:**
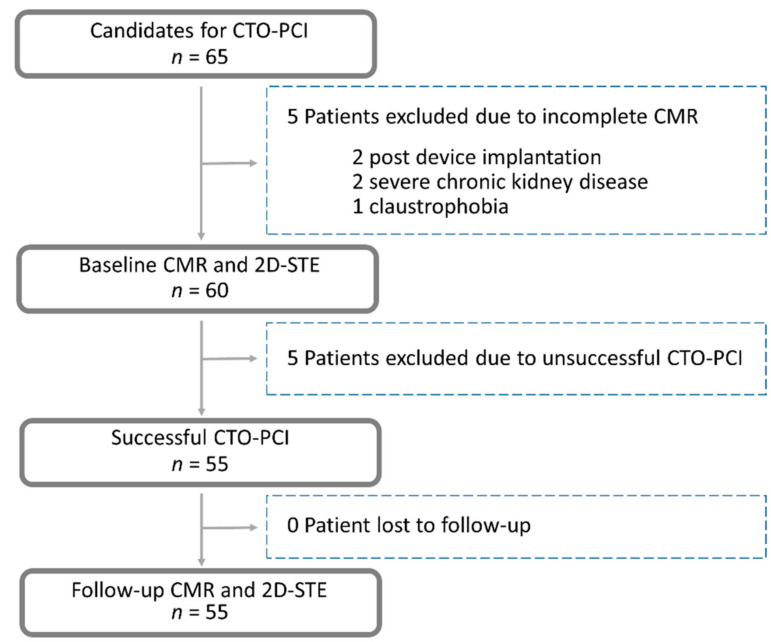
Flow chart of patient enrollment. Abbreviations: CAG, coronary angiography; CMR, cardiovascular magnetic resonance; CTO, chronic total occlusion; PCI, percutaneous coronary intervention; 2D-STE, two-dimensional speckle-tracking echocardiography.

Of the 65 patients initially recruited, 55 completed the study (82% men; mean age 69 ± 10 years) (Figure 1). Five patients were excluded because of incomplete MRI data and five because of CTO-PCI technical failure. No patient was lost to follow-up. There was no acute or subacute stent thrombosis, death, spontaneous acute myocardial infarction, emergent re-PCI, emergent coronary artery bypass graft surgery, or acute stroke during the follow-up.

Patient characteristics are shown in Table 1. Medication use was similar at baseline and follow-up. There was no statistical difference between study-completion patients and excluded patients.

Angiographic and procedural characteristics of the patients are shown in Table 2. The CTO was in the left anterior descending artery in 11 (20%) patients, right coronary artery in 33 (60%), and circumflex artery in 11 (20%). There was no patient with different vessel CTOs eligible for PCI. The retrograde approach was used in 30 (55%) patients.

### 3.2. Transmural Extent of LGE MRI

From baseline to follow-up, the enhanced area percentage did not change. All 880 segments were evaluable for the presence/absence of late enhancement by LGE MRI. Among the 284 segments of the CTO territory, 92 (32.4%) had no late enhancement (normal), 130 (45.8%) showed delayed enhancement with transmural extent of 1–50%, and 62 (21.8%) showed delayed enhancement with a transmural extent of 51–100%.

### 3.3. Myocardial Perfusion Reserve

**Figure 2:**
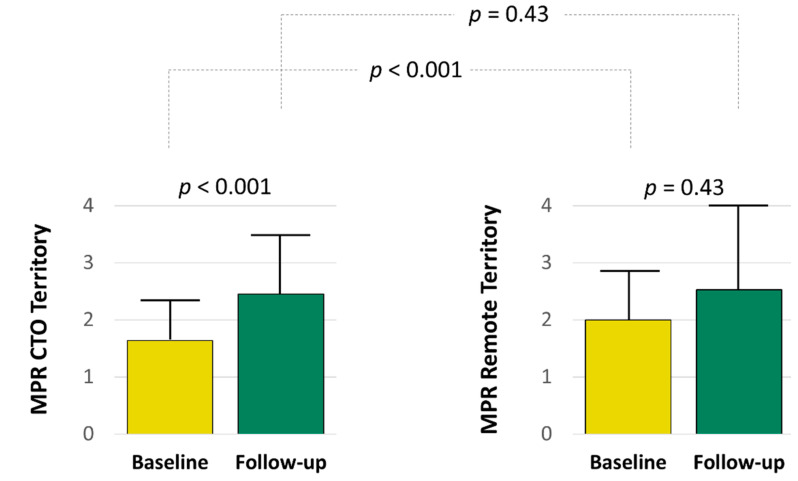
Cardiovascular magnetic resonance findings: myocardial perfusion reserve. Abbreviations: CTO, chronic total occlusion; MPR, myocardial perfusion reserve.

At baseline, MPR in the CTO territory was lower than that in a remote territory (1.64 ± 0.64 vs. 2.00 ± 0.89, *p* < 0.001) (Figure 2), but it improved significantly after revascularization (1.64 ± 0.64 vs. 2.45 ± 1.07, *p* < 0.001). MPR after revascularization was similar between the CTO territory and a remote territory (2.45 ± 1.07 vs. 2.53 ± 1.48, *p* = 0.43). There were no differences in the MPR in the remote territory before and after PCI.

### 3.4. Changes in Echocardiographic Parameters

Table 3 shows echocardiographic data at baseline and follow-up. From baseline to follow-up, there was a significant increase in the LV ejection fraction (from 54.3% ± 12.2% to 58.2% ± 9.1%, *p* = 0.004). Following CTO-PCI, the global LS (−16.60% ± 3.79% to −18.61% ± 3.29%, *p* < 0.001), global CS (−15.92% ± 3.44% to −18.61% ± 3.29%, *p* < 0.001), and wall motion score (WMS) index (1.35 ± 0.52 to 1.24 ± 0.36, *p* = 0.006) were significantly improved.

### 3.5. Improvement in Segmental LV Function Stratified by LGE MRI and MPR

**Figure 3:**
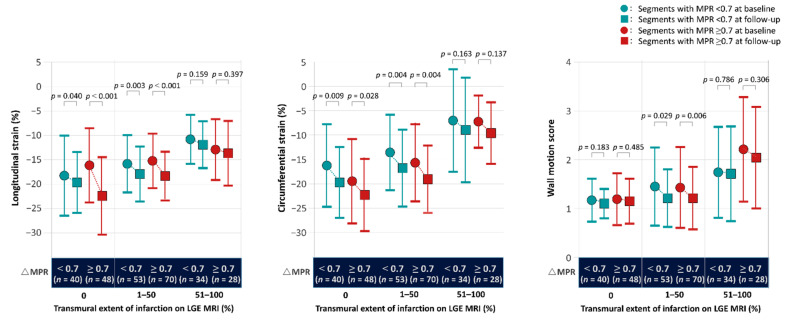
Changes in strain parameters stratified by %LGE and changes in MPR from baseline to follow-up. Abbreviations: LGE, late gadolinium enhancement; MPR, myocardial perfusion reserve; MRI, magnetic resonance imaging.

Figure 3 shows the changes in echocardiographic parameters in segments in the territory of the recanalized artery during follow-up, stratified by the transmural extent of LGE MRI (0%, 1–50%, >50%) and changes in MPR from baseline to follow-up (≥0.7 (median value) or <0.7). Segments with more negative peak systolic strain or lower WMS belonged in a lower hyper-enhancement category at baseline and follow-up. In the CTO territory, regardless of MPR changes, the LS and CS were significantly improved in segments with a transmural extent of LGE ≤ 50% but not in those with a transmural extent of LGE > 50%. WMS was only significantly improved in segments with a transmural extent of LGE of 1–50%.

### 3.6. ROC Curves of Echocardiographic Parameters to Identify Segments with LGE > 50%

**Figure 4:**
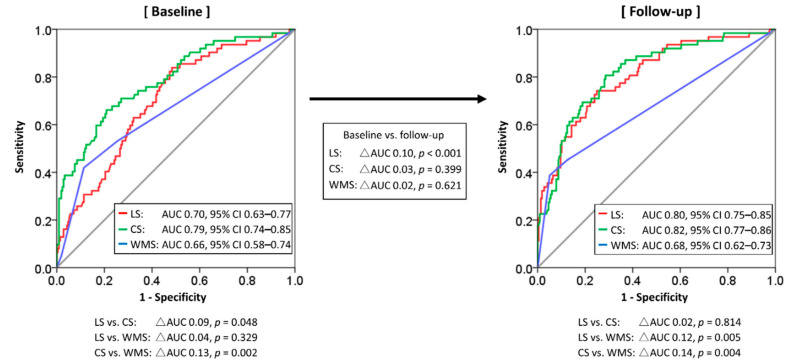
Comparisons of discriminatory accuracy of echocardiographic parameters at baseline and follow-up for nonviable segments in LGE MRI images prior to the procedure. Abbreviations: AUC, area under the curve; CS, circumferential strain; LS, longitudinal strain; WMS, wall motion score.

Figure 4 shows the ROC curves of echocardiographic parameters at baseline and follow-up for identifying segments with a transmural extent of LGE > 50% in the perfusion territory of the CTO vessel. At baseline, CS allowed the identification of segments of LGE > 50% with a higher accuracy than LS (AUC 0.79 vs. 0.70, *p* = 0.048) or WMS (AUC 0.79 vs. 0.66, *p* = 0.002). With a cutoff value of −10.7, the sensitivity, specificity, and accuracy of pre-procedural CS for detecting segments with LGE > 50% were 71%, 73%, and 79%, respectively. The discriminatory ability of LS for segments with a transmural extent of LGE >50% was significantly improved during the follow-up period after successful CTO-PCI in the recanalized artery territory (AUC 0.70 (95% confidence interval 0.63–0.77) vs. 0.80 (0.75–0.85), *p* < 0.001), whereas that of CS or WMS did not change from baseline to follow-up. At follow-up, CS identified segments of LGE > 50% better than WMS (AUC 0.82 vs. 0.68, *p* = 0.004) but not better than LS (AUC 0.82 vs. 0.80, *p* = 0.814).

### 3.7. Multiple Linear Regression Analyses

Table 4 and Table 5 show multiple linear regression analyses of the relationships between changes in strain parameter, %LGE, and changes in MPR from baseline to follow-up. The transmural extent of the LGE effect was significantly associated with changes in LS and CS from baseline to follow-up. In contrast, changes in MPR from baseline to follow-up were significantly associated with changes in LS but not CS.

### 3.8. Reproducibility of Strain Measurements

Intra-observer and intra-class correlation coefficients for LS and CS were 0.93 and 0.92 respectively, and the inter-observer and intra-class correlation coefficients were 0.86 and 0.84, respectively.

## 4. Discussion

There were three major findings of this study: (1) With a cutoff value of −10.7, pre-procedural CS showed reasonable sensitivity (71%), specificity (73%), and accuracy (79%) for detecting segments with transmural scar. (2) The discriminatory ability of LS for segmental scar transmurality assessment improved significantly in the territory of the recanalized artery during follow-up after successful CTO-PCI. The accuracy of LS was lower than that of CS at baseline, but increased at follow-up. (3) Reduction of the myocardial ischemic burden using CTO-PCI was significantly associated with changes in LS (but not in CS) from baseline to follow-up.

In line with previous reports, the CS assessment allowed the better differentiation of segments with transmural scar than LS before CTO-PCI, indicating the greater value of using CS for preprocedural assessment of CTO-PCI indications. In contrast, LS was sensitive to the reduction in ischemic burden by CTO-PCI, suggesting that it might be helpful for monitoring the effect of CTO revascularization. The differences in utility among strain parameters could be partly explained by differences in the sensitivity to the myocardial ischemic burden.

### 4.1. Strain Parameters as a Surrogate for Viability to Aid the Appropriate Patient Selection for CTO-PCI

CS has shown a greater ability than LS to differentiate segments with transmural or non-transmural scars, via a mechanism related to the function of subepicardial and mid-wall myocardial fibers [15,17]. If the myocardial infarction extends beyond the subendocardial layer and reaches the mid-wall, CS impairment becomes apparent. Our results were in line with those reports [18,19], and the cutoff value of CS was similar to that of −10.5 reported previously [18].

### 4.2. Strain Parameters for the Detection of Left Ventricular Functional Improvement by CTO-PCI

In the present study, changes in quantitative measurements of the myocardial ischemic burden from baseline to follow-up were significantly associated with changes in the LS but not in the CS. Moreover, the discriminatory ability of LS for segmental scar transmurality assessment significantly improved during follow-up after successful CTO-PCI in the perfusion territory of the recanalized artery, and increased to a level similar to that of CS during the follow-up. In contrast, the discriminatory ability of CS did not change from baseline to follow-up. LS is the most sensitive strain parameter to detect subtle impairments of contractile function in coronary artery disease [20,21] and other diseases [22,23,24]. Therefore, the findings of LS in the current study, including the inferior discriminatory ability of LS for transmural scars at baseline, improvement of discriminatory ability at follow-up, and the significant association between changes in LS and those in MPR, might have been caused in part by the high sensitivity of LS for ischemic status. Global LS has been shown to provide prognostic information in a wide range of myocardial diseases [25]. Therefore, by using LS for evaluation of treatment effects, clinical advantages conferred by successful CTO-PCI could be implied, even in this study cohort with considerably preserved contractile function.

### 4.3. Clinical Implications

With CTO-PCI gaining wider acceptance as a therapeutic option for coronary artery disease, the importance of valid patient selection has increased. On the basis of the recent increase in the importance of physiological assessment for coronary artery disease, quantitative measurements of the myocardial ischemic burden are frequently performed before coronary interventions. However, it would not be practical to manipulate a pressure wire to the distal sites of CTO lesions for preprocedural assessment for indications of CTO-PCI. Approximately 40% of CTO patients have had a previous myocardial infarction, a rate twice that of patients without CTO lesions [26]. Furthermore, the distribution of these infarctions has commonly been reported to be inhomogeneous with the total wall thickness.

With its excellent spatial resolution, cardiovascular MRI provides detailed, segmental, quantitative analyses of myocardial ischemia and scar transmurality. However, it also has limitations, including contraindications, cost, accessibility, and side effects from the contrast medium or adenosine administration. We previously showed that the optimal cut-off value of the extent of LGE MRI was 50% for functional recovery after CTO-PCI [27]. In the previous retrospective study, LGE MRI was performed one time only, at 8 ± 2 months after CTO-PCI, and the ischemic burden was not measured quantitatively. Meanwhile, in the current prospective study, LGE MRI and MPR were serially obtained prior to and 8 ± 2 months after CTO-PCI. Between the previous study and the current one, there was no overlap in the inclusion period or in the included patients undergoing CTO-PCI.

PCI is rapidly becoming a valid option for complex high-risk (and indicated) patients [28]. Some contraindications for cardiovascular MRI—such as after implantation of a defibrillator or other metallic devices, chronic kidney disease for contrast use, and conduction disorders for adenosine stress tests—are frequently present in patients undergoing CTO-PCI who have been referred because of surgical ineligibility. Therefore, establishing a non-invasive method for CTO-PCI patient selection, especially those contraindicated for cardiovascular MRI, is urgently needed. We showed that 2D-STE might overcome these limitations of cardiovascular MRI, enabling the non-invasive evaluation of patients with CTO.

### 4.4. Limitations

There were several limitations in this study. First, it was a single-center, observational study. Second, myocardial strain could not be automatically analyzed via echocardiography because of poor image quality in 5.6% of the segments. However, this value is comparable to those of previous reports. Third, the rate of non-LAD-CTO was 80% in the current study. Accordingly, it would be challenging to show a prognostic benefit conferred by successful CTO-PCI in this study cohort with relatively preserved contractile function. However, according to guidelines for stable coronary artery disease, LAD-CTO impacts the selection of treatment strategies, especially for patients with multivessel disease, decreasing the likelihood of PCI. This is a bias inherent in observational studies. Moreover, we think that improving survival is not the only potential benefit of CTO-PCI. Improvements also exist related to clinical outcomes, including improved left ventricular systolic function and significant reductions in angina burden after CTO-PCI. These potential benefits may also justify attempted CTO-PCI despite a lack of significant improvements in survival. Fourth, the rate of the retrograde approach in the current study was relatively high in comparison to recently standardized procedures. Some universal algorithms regarding CTO-PCI strategy were recently developed [5,29]. However, during the inclusion period of the current study, the selection of the procedural strategy for CTO-PCI was at the operator’s discretion and this differed between each operator. In the present study, the retrograde approach was used aggressively to secure the distal lumen beyond the CTO lesion, based on a high re-occlusion rate of the subintimal tracking and re-entry technique reported previously [30]. Fifth, the echocardiographic finding of an end-diastolic wall thickness < 6 mm was not included in the current study. This finding was reported to indicate a lack of contractile reserve and functional recovery after revascularization [31]. In the current study, among 284 segments in the perfusion territory of the CTO vessel, only 12 segments (4%) showed an end-diastolic wall thickness < 6 mm, which might be caused by the strict criteria including patients with symptomatic angina and/or positive for ischemia. As shown in Supplementary Table S1, none of the echocardiographic contractile parameters changed significantly in segments with an end-diastolic wall thickness < 6 mm. Sixth, although not used in the current study due to restricted accessibility, T1 mapping might provide incremental value by quantifying focal and diffuse alterations in myocardial structure not assessable by LGE [32]. Actually, extracellular volume fraction, which reflects the size of the extracellular space in the myocardium and correlates with the histological measurements of the extracellular matrix, is a better predictor of functional recovery following revascularization compared with the transmural extent of scars. Seventh, although not performed in the current study, an additional layer-specific speckle-tracking analysis might improve the accuracy of the current analysis [33]. Thus, further study is needed to elucidate whether non-invasive assessment using 2D-STE contributes to better outcomes in CTO patients.

## 5. Conclusions

Although cardiovascular MRI, which allows segmental and quantitative analyses of myocardial ischemia and scar transmurality, is useful for assessment of CTO patients, it has limitations, including contraindications, cost, and accessibility. However, 2D-STE may overcome these limitations for cardiovascular MRI. Application of 2D-STE for CTO-PCI might noninvasively provide appropriate patient selection, sensitive and objective information regarding clinical benefits conferred by successful results, and wider use of the treatment procedure in clinical practice.

## Figures and Tables

**Table 1:** Patient characteristics.

	Patients (*n* = 55)
Age, years	69 ± 10
Male gender	45 (82%)
Dyslipidemia	45 (82%)
Diabetes mellitus	19 (35%)
Hypertension	45 (73%)
Past smoking	36 (65%)
History of previous myocardial infarction	25 (45%)
History of coronary artery bypass grafting	4 (7%)
NYHA I	41 (75%)
II	10 (18%)
III	4 (7%)
IV	0 (0%)
CCS 0	7 (12%)
I	13 (22%)
II	19 (32%)
III	13 (22%)
IV	3 (5%)
Chronic obstructive pulmonary disease	11 (19%)
eGFR, mL/min/1.73 m^2^	69.3 ± 15.3
Medical treatments	
Antiplatelet drugs	55 (100%)
β-blockers	44 (80%)
ACE-inhibitors/ARB	44 (80%)
Statins	55 (100%)

Values are presented as mean ± standard deviation or as number (percentage). ACE, angiotensin-converting enzyme; ARB, angiotensin receptor blocker; CCS, Canadian Cardiovascular Society; eGFR, estimated glomerular filtration rate; NYHA, New York Heart Association.

**Table 2:** Angiographic and procedural characteristics.

	Patients (*n* = 55)
CTO vessel	
Left anterior descending	11 (20%)
Left circumflex	11 (20%)
Right	33 (60%)
J-CTO score	1.56 ± 1.27
Entry shape—blunt	20 (36%)
Calcification	16 (30%)
Bending > 45°	26 (47%)
Occlusion length ≥ 20 mm	21 (38%)
Reattempted lesion	5 (9%)
Reference diameter, mm	2.8 ± 0.8
Occlusion length, mm	25 ± 21
Collateral connection grade	
CC0	9 (16%)
CC1	29 (53%)
CC2	17 (31%)
Antegrade approach only	25 (45%)
Retrograde approach	30 (55%)
Antegrade wiring techniques	
Parallel wire technique	21 (38%)
IVUS-guided wiring technique	4 (7%)
Successfully crossed collateral channel in retrograde approach	
Septal channel	22 (73%)
Epicardial channel	5 (17%)
Bypass graft	3 (10%)
Successful CTO crossing strategy in retrograde approach	
Reverse CART	15 (50%)
Retrograde wire cross	10 (33%)
Kissing wire cross	4 (13%)
CART	1 (3%)

Values are presented as mean ± standard deviation or as number (percentage). CART, controlled antegrade and retrograde subintimal tracking; CC, collateral channel; CTO, chronic total occlusion; IVUS, intravascular ultrasonography.

**Table 3:** Changes in echocardiographic parameters from baseline to follow-up.

	Baseline	Follow-Up	*p*-Value *
LVEF, %	54.3 ± 12.2	58.2 ± 9.1	0.004
GLS, %	−16.60 ± 3.79	−18.61 ± 3.29	<0.001
GCS, %	−15.92 ± 3.44	−18.04 ± 3.69	<0.001
WMSI	1.35 ± 0.52	1.24 ± 0.36	0.006

Values are presented as means ± standard deviation. Statistically significant values (*p* < 0.05) are highlighted in bold. EDV, end-diastolic volume; ESV, end-systolic volume; GCS, global circumferential strain; GLS, global longitudinal strain; LVEF, left ventricular ejection fraction; WMSI, wall motion score index. * *p*-value represents the significance of change in each parameter from baseline to follow-up.

**Table 4:** Multiple linear regression analysis of the relations between changes in longitudinal strain, transmural extent of late gadolinium enhancement, and changes in myocardial perfusion reserve.

	β	SE	95% CI for β	*p*-Value
%LGE at baseline	0.033	0.011	0.012 to 0.054	0.002
Changes in MPR from baseline to follow-up	−1.103	0.257	−1.608 to −0.597	<0.001

LGE, late gadolinium enhanced; MPR, myocardial perfusion reserve.

**Table 5:** Multiple linear regression analysis of the relations between changes in circumferential strain, transmural extent of late gadolinium enhancement, and changes in myocardial perfusion reserve.

	β	SE	95% CI for β	*p*-Value
%LGE at baseline	0.047	0.019	0.009 to 0.085	0.017
Changes in MPR from baseline to follow-up	0.426	0.467	−0.492 to 1.345	0.362

LGE, late gadolinium enhanced; MPR, myocardial perfusion reserve.

## Data Availability

Data are available upon request to the corresponding author.

## Supplementary Materials

The following are available online at https://www.mdpi.com/article/10.3390/jcm10204712/s1, Table S1: Echocardiographic parameters of contractile function for segments with end-diastolic wall thickness < 6 mm at baseline and follow-up.
