# Supplementary material for: Non-Invasive Evaluation of Patients Undergoing Percutaneous Coronary Intervention for Chronic Total Occlusion
Source: J Clin Med. 2021 Oct 14;10(20):4712. doi: 10.3390/jcm10204712 (PMC8541262; doi:10.3390/jcm10204712)
Supplement: Supplementary file 1 [file jcm-10-04712-s001.zip › jcm-1397896-supplementary.pdf]

Journal of
Clinical Medicine

MDPI

Supplementary materials

Table S1. Echocardiographic parameters of contractile function for segments with end-diastolic wall thickness <6mm at baseline and follow-up.

	Baseline	Follow-up	P value
LS, (%)	-7.43±3.25	-7.95±3.06	0.67
CS, (%)	-4.47±3.60	-4.33±7.59	0.94
WMS	2.75±1.06	2.83±0.94	0.77
